# Advances of two-stage riser catalytic cracking of heavy oil for maximizing propylene yield (TMP) process

**DOI:** 10.1007/s13203-014-0086-6

**Published:** 2014-09-25

**Authors:** Yang Chaohe, Chen Xiaobo, Zhang Jinhong, Li Chunyi, Shan Honghong

**Affiliations:** State Key Laboratory of Heavy Oil Processing, China University of Petroleum, Qingdao, 266580 Shandong China

**Keywords:** Two-stage riser, Catalytic cracking, Propylene

## Abstract

Two-stage riser catalytic cracking of heavy oil for maximizing propylene yield (TMP) process proposed by State Key Laboratory of Heavy oil Processing, China University of Petroleum, can remarkably enhance the propylene yield and minimize the dry gas and coke yields, and obtain high-quality light oils (gasoline and diesel). It has been commercialized since 2006. Up to now, three TMP commercial units have been put into production and other four commercial units are under design and construction. The commercial data showed that taking paraffinic based Daqing (China) atmospheric residue as the feedstock, the propylene yield reached 20.31 wt%, the liquid products yield (the total yield of liquefied petroleum gas, gasoline, and diesel) was 82.66 wt%, and the total yield of dry gas and coke was 14.28 wt%. Moreover, the research octane number of gasoline could be up to 96.

## Introduction

As the development of economy, the demand for propylene and ethylene is growing rapidly. The naphtha steam cracking process has been the major source of light olefins for more than half a century [1]. It can provide over 90 % of ethylene and approximately 65 % of propylene in global market [2]. In recent years, because of the shortage of naphtha, the cheaper raw materials, such as ethane and shale gas, are used as feedstocks of steam cracking. Consequently, the P/E (propylene to ethylene) ratio in the production of steam cracking reduced. Nevertheless, in the present market, the demand growth rate of propylene is even higher than that of ethylene. Therefore, the heavy oil catalytic cracking/pyrolysis process, which can not only reduce the energy consumption, but also increases the P/E ratio, has become the important supply of propylene. In comparison to the naphtha steam cracking process, the heavy oil catalytic cracking/pyrolysis process has obvious advantages, such as abundant feedstocks, low cost, mild operating conditions, low energy consumption, and so on.

To produce more propylene from heavy oil catalytic cracking process, there are two pathways. One is the addition of additives in the conventional FCC reaction-regeneration system, and the yield of propylene rises generally by 30–40 % depending on the process and conditions when the additive accounts for 3–5 % of the catalyst inventory in the system [3–6]. Another way is developing special FCC processes. Nowadays, heavy oil catalytic cracking/pyrolysis process for light olefins has become an important objective of oil companies all over the world. The SINOPEC developed processes of maximizing gaseous olefins and gasoline with atmospheric residue (ARGG) [7, 8], deep catalytic cracking (DCC) [9, 10], a catalytic cracking process for the production of clean gasoline (MIP-CGP) [11, 12], and the UOP, AXENS, and SHELL companies developed the PetroFCC, PetroRiser, and MILOS processes, respectively. The yield of propylene in these processes can up to 10–20 wt% and much more than that of conventional FCC. These processes share the following common characteristics: multi-reaction zones and/or high operation severity. For the DCC and MIP-CGP processes, there is a diameter-enlarged stage in the middle of the riser; for MILOS and PetroFCC, there are two risers: one feeds conventional feedstock and another is specially for cracking FCC gasoline [6]. However, the major problem for these processes is that, high propylene yield always with high dry gas yield and inferior quality of light oils (gasoline and diesel). State Key Laboratory of Heavy Oil Processing, China University of Petroleum, proposed a novel process for maximizing propylene yield by two-stage riser catalytic cracking of heavy oil (called TMP for short). The TMP process has four advantages: maximizing the yield of propylene, enhancing the octane number of gasoline, high-quality light cycle oil (diesel fraction), and low dry gas and coke yields. The present paper introduces the details of experimental results and commercial application of TMP process.

## Two-stage riser catalytic cracking of heavy oil for maximizing propylene yield (TMP)

**Fig. 1:**
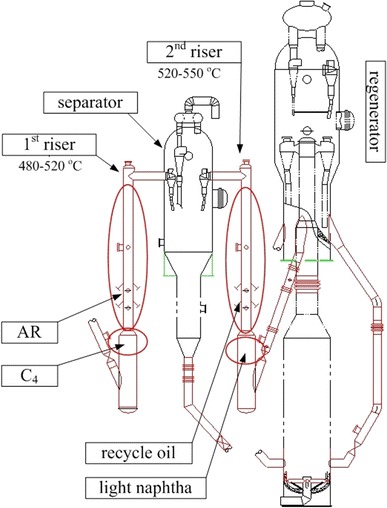
Schematic diagram of the TMP process

To improve the yield of propylene from heavy oil catalytic cracking/pyrolysis with low dry gas yield and high-quality light oils, the novel process should solve the following challenges: (a) restraining thermal cracking to minimize dry gas yield; (b) reducing the paraffin content of liquefied petroleum gas (LPG); (c) increasing the aromatic content of gasoline and decreasing its paraffin and olefin content; (d) decreasing the over-cracking of diesel to keep its quality. Figure 1 shows the schematic diagram of the TMP process (the reaction-regeneration system).

In the first riser, butenes (C_4_) and the fresh FCC feed are fed by stratified injections; in the second riser, the light gasoline (mainly composed of C_5_ and C_6_ olefins) and the recycling oil are fed by the same manner. The regenerated catalyst firstly contacts with C_4_ (in the first riser) and light naphtha (in the second riser), then reacts with atmospheric residue (AR) and recycling oil, respectively. According to the characteristics of the above four feedstocks, the proper conditions may be compromised. For instance, the reaction zones of C_4_ and light naphtha at the bottom of the two risers need a high catalyst density and a short residence time. The outlet temperature of the first riser is commonly operated between 480 and 520 °C, while that for the second riser is 520–550 °C, only slightly higher than the conventional FCC process.

The TMP process optimizes two riser reactors to gain the flexibility of operation; develops a proprietary catalyst with low hydrogen transfer activity and high cracking activity; combines stratified injections of light and heavy feedstocks and specially designed reactors to obtain high catalyst to oil ratio (C/O ratio) and low reaction temperature in riser exits. These effective methods can well meet the above challenges and remarkably enhance the yield of propylene with low dry gas and coke yields.

- Developing a proprietary catalyst with low hydrogen transfer reaction activity and high heavy oil cracking activity to remarkably enhance the yield and selectivity of propylene and the feedstock conversion.
- Selectively recycling C_4_ and light naphtha to the bottom of the first riser and the second riser, respectively. On one hand, the C_4_ and light naphtha can crack with higher operating severity to enhance the conversion of C_4_ and light naphtha into propylene. Meanwhile, the olefin content of gasoline can be reduced. On the other hand, as the injection of C_4_ and light naphtha, because of the energy of evaporation and reaction, the regenerated catalyst should provide more heat to the riser reactor. According to the heat balance, it requires a larger catalyst circulation. The higher the catalyst circulation is, the larger the C/O ratio is. Thus, the conversion of C_4_, light naphtha, and heavy oil can be enhanced.
- TMP process proposes a novel reactor with higher catalyst density. On the basis of the previous studies [13, 14], it was found that the solid catalyst density in the reactor can have a significant increase with an enlarged section of riser. Therefore, TMP process proposes a novel reactor with higher catalyst density, which can enhance the contact efficiency between oil vapor and catalyst to further increase the conversion of C_4_ and light naphtha.
- Shortening the residence time of oil vapor in the risers. In previous work, it was found that propylene was also an intermediate product, thus a relatively short reaction time should be taken to avoid the further conversion of propylene. Zhang et al. [15] also found that when operated under a shorter residence time, more diesel with higher quality could be obtained.

In the catalytic pyrolysis process of heavy oil, how to reduce the dry gas and coke yields is an acknowledged challenge, as that kind of process is commonly operated at high operating severity. It has been well-established that both thermal cracking and catalytic cracking can generate dry gas. For thermal cracking, free radical chain reaction, which has much higher activation energy than catalytic cracking, is the main mechanism [16]. Haag and Dessau [17] proposed that H_2_, CH_4_ and C_2_ hydrocarbons also could be created by monomolecular proteolytic cracking route, and found that the activation energy for the proteolytic cracking was higher than that for the β-scission. Thus both the thermal cracking and the monomolecular cracking favored higher temperature. In the TMP process, on injection of C_4_ and light naphtha, the C/O ratio can be increased significantly without increasing the riser outlet temperature, and higher feed conversion and propylene yield can be achieved. Thus, the ratio of thermal cracking and monomolecular cracking can be reduced. Moreover, after the reaction of C_4_ and light naphtha, the temperature of catalyst is lowered significantly, but the activity can remain a high level, thus the thermal cracking of heavy oil can be restrained. On the other hand, the injection of heavy oil can control the high temperature reactions of C_4_ and light naphtha at a proper reaction time, and restrains the thermal cracking of C_4_ and light naphtha. In addition, the increased catalyst density in the novel reactor also can enhance the catalytic cracking of C_4_ and light naphtha, and help to reduce the dry gas and coke yields. Consequently, the TMP process can enhance the propylene yield and restrain the dry gas and coke yields.

## Experimental study of TMP process

### Effect of catalyst on propylene yield

**Fig. 2:**
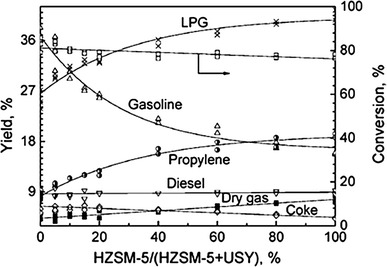
Effect of catalyst on product distribution

Generally, HZSM-5 is the optimum zeolite in producing propylene; its ability to crack heavy oil, however, is very weak. Therefore, a certain amount of Y or USY zeolite must be added in the catalyst to ensure the conversion of heavy oil. Figure 2 shows that a certain amount of HZSM-5 in the catalyst system is essential for increasing the propylene yield. The rising rate of the propylene yield first increased fast, and then slowed down when the ratio of HZSM-5 exceeds 60 % [9]. By contrast, the feedstock conversion decreased as the increasing of HZSM-5 ratio. Therefore, the ratio of HZSM-5/USY (or Y) should be proper to ensure the conversion of heavy oil.

### Stratified injections of C_4_ and AR

In the first stage riser, the C_4_ is injected to the bottom of the riser and first reacted with the regenerated catalyst under higher operating severity, then the AR is injected and reacts in the presence of the catalyst at lower temperature. The experimental results listed in Table 1 show that under the stratified injection mode, in comparison to the calculated results, the dry gas yield reduced by about 40 % and the liquid products yield increased 2.45 wt%. The injection of C_4_ only slightly influenced the AR conversion, but the C_4_ can be further converted into propylene without increasing the dry gas yield significantly.

**Table 1:** Comparison of FCC product distributions between the stratified injection of C_4_ and AR in the first stage riser and their respective reaction process [9]

Items	C_4_ + AR	AR	C_4_	Calculation^a^	Δ^b^
Mass ratio	16.26:100				
Reaction temperature (°C)	510	510	510		
Catalyst/oil (kg/kg)	8.5	7	8		
Residence time (s)	1.21	1.38	1.45		
Product distribution (wt%)					
Dry gas	3.85	3.70	15.71	6.25	−2.40
LPG	33.13	35.98	65.76	30.41	2.72
Gasoline	27.05	25.04	14.75	27.44	−0.39
Diesel	14.52	14.40	0.00	14.40	0.12
Heavy oil	15.23	15.03	0.00	15.03	0.20
Coke	6.22	5.85	3.78	6.46	−0.24
Light oil yield	41.57	39.44	14.75	41.84	−0.27
Liquid products yield	74.70	75.42	80.51	72.25	2.45
Conversion	84.77	84.97	–	84.97	−0.20
Ethylene	2.77	2.65	7.35	3.85	−1.08
Propylene	18.63	16.44	28.65	21.10	−2.47
Butenes	9.80	16.26	22.94	3.73	6.07

^a^For LPG and butenes yield, $y=y_{II}+y_{III}\cdot 16.26\;-16.26\;\%$, for other products yield, $y=y_{II}+y_{III}\cdot 16.26\;\%$, where, the $y_{II}$ and $y_{III}$ mean yield from data column II and III, respectively

^b^Item Δ is the values of stratified injection minus that of calculation

### Stratified injections of light naphtha and heavy cycle oil (HCO)

In the second stage riser, the recycle ratio of light naphtha reached to 83 %, compared to HCO feed; therefore, after the reaction of light naphtha, the temperature of catalyst would decrease sharply. Moreover, HCO is more difficult to be cracked than the fresh feedstock. Thus, the reaction temperature of the second stage riser was higher than the first one. The stratified injection results of the light naphtha and the HCO according to the ratio of their yield in the first stage riser are listed in Table 2. In comparison to the calculated results, the yield of dry gas under the stratified injection mode decreased by around 32 %, and the yield of liquid products increased by approximately 10 %. In addition, after the reaction of light naphtha, most of the olefins were converted into light olefins or other hydrocarbons, such as isoparaffins, aromatics, etc. Thus, the olefin content of gasoline can be reduced without octane loss.

**Table 2:** Comparison of FCC product distributions between the stratified injection of light naphtha and HCO in the second stage riser and their respective reaction process [9]

Items	Light naphtha + HCO	HCO	Light naphtha	Calculation^a^	Δ^b^
Mass ratio	12.68:15.23				
Reaction temperature (°C)	530	530	530		
Catalyst/oil (kg/kg)	10.5	8.5	9.5		
Residence time (s)	1.85	1.93	1.72		
PRODUCT distribution (wt%)					
Dry gas	8.30	4.44	21.76	12.31	−4.01
LPG	39.63	24.75	44.03	33.51	6.12
Gasoline	23.15	16.37	30.33	22.71	0.44
Diesel	8.57	16.26	0.00	8.87	−0.30
Heavy oil	16.27	33.43	0.00	18.24	−1.97
Coke	4.07	4.74	3.88	4.35	−0.28
Light oil yield	31.72	32.63	30.33	31.59	0.13
Liquid products yield	71.35	57.38	74.36	65.09	6.26
Conversion	83.73	66.57	–	81.76	1.97
Ethylene	6.84	2.76	16.00	8.78	−1.94
Propylene	19.65	12.39	26.68	18.88	0.77
Butenes	15.22	9.89	12.46	11.06	4.16

^a^For products yield, $y=(y_{II}\cdot 15.23\;\%+y_{III}\cdot 12.68\;\%)/27.91\;\%$_,_ where, the $y_{II}$ and $y_{III}$ mean yield from data column II and III, respectively

^b^Item Δ is the values of stratified injection minus that of calculation

## The industrial application of the TMP process

In 2006, the TMP technology was firstly applied in a 120 kt/a industrial unit belonging to CNPC Daqing Refining and Chemical Branch Co. Using paraffinic based Daqing atmospheric residue (density (20 °C) was 900 kg/m^3^; Conradson carbon residue was 4.6 wt%; the content of carbon was 86.00 wt%; and the content of hydrogen was 12.78 wt%) as the feedstock, the propylene yield reached 20.31 wt%, the liquid products yield was 82.66 wt%, and the total yield of dry gas and coke was 14.28 wt%. Moreover, the olefin volume fraction of gasoline was 32 %, and the research octane number of gasoline could be up to 96. The density (at temperature 20 °C) of diesel was 910 kg/m^3^.

At present, three TMP commercial units have been put into production and other four commercial units are under design and construction.

## Conclusion

Developing the special FCC processes for maximizing propylene always meets a baffling problem which is that, high propylene yield always with high dry gas yield and inferior quality of light oils. The TMP process has four effective measures to solve this challenge: (a) developing a proprietary catalyst with low hydrogen transfer reaction activity and high heavy oil cracking activity; (b) combining stratified injections of light and heavy feedstocks and specially designed reactors; (c) designing a novel reactor with higher catalyst density; (d) shortening the residence time of oil vapor in the risers. The experimental study and commercial application verified that the TMP process can remarkably enhance the propylene yield and minimize the dry gas and coke yields, and meanwhile, obtain high-quality light oils. At present, three TMP commercial units have been put into production and other four commercial units are under design and construction. The commercial data showed that taking paraffinic based atmospheric residue as the feedstock, the propylene yield reached 20.31 wt%, the liquid products yield was 82.66 wt%, and the total yield of dry gas and coke was 14.28 wt%. Moreover, the olefin volume fraction of gasoline was 32 %, and the research octane number of gasoline could be up to about 96.
